# Supplementary material for: Monsters with a shortened vertebral column: A population phenomenon in radiating fish Labeobarbus (Cyprinidae)
Source: PLoS One. 2021 Jan 20;16(1):e0239639. doi: 10.1371/journal.pone.0239639 (PMC7817016; doi:10.1371/journal.pone.0239639)

**SUPPLEMENT list to Golubtsov et al.**

**S1 Table.** Occurrence of aberrant individuals or stocks with shortened vertebral column and abnormally short and deep body in the different groups of fish. (DOCX)

**S1 Fig.** Photographs of two habitats at the main sampling (no. 1) of the middle Genale River – (A) the continuous pool and (B) the upper section of rapids downstream of the pool. (DOCX)

**S2 Table.** Size characteristics of the cast and gill net catches for different *Labeobarbus* forms from the middle Genale assemblage sampled in 2009 and 2019. Size characteristics of the aged individuals from the gill net catches of 2019. (DOCX)

**S3 Table**. Sample size of subsets analyzed in study. (DOCX)

**S1 Supporting material**. Data on effect size of samples for Kruskal-Wallis test (A) and Mann-Whitney U test (B). (DOCX)

**S2 Fig.** Two piscivorous barbs with normal and deformed vertebral column. (DOCX)

**S4 Table**. Gill net catch composition and percentage of different *Labeobarbus* morphs in the middle Genale assemblage in 2009 and 2019. (DOCX)

**S5 Table.** Eigenvectors of 10 most loaded characters to Fig. 5A. (DOCX)

**S6 Table.** Descriptive statistics of vertebral counts. (DOCX)

**S3 Fig.** An addition to Fig. 8. Distribution of deformed vertebrae along vertebral column in individuals with 40 and 42 total vertebrae. (DOCX)

**S4** **Fig.** Growth of short (SH), generalized (GN) and lipped (LP) forms in the middle Genale *Labeobarbus* assemblage in sample of 2009 estimated with standard length (SL). (DOCX)

**S1 Table.** Occurrence of aberrant individuals or stocks with the shortened vertebral column and abnormally short and deep body in the different groups of fish.

| **Family, species** | **Manifestation** | **Source** |
| --- | --- | --- |
| Anguillidae | | |
| European eel, *Anguilla anguilla* L. 1758 | the only 8-year female from Iceland | Wunder (1968) |
| Clupeidae | | |
| Atlantic menhaden, [*Brevoortia*](http://researcharchive.calacademy.org/research/ichthyology/catalog/fishcatget.asp?genid=2769) [*tyrannus*](http://researcharchive.calacademy.org/research/ichthyology/catalog/fishcatget.asp?spid=60556) (Latrobe [1802)](http://researcharchive.calacademy.org/research/ichthyology/catalog/getref.asp?id=17488) | three hump-backed individuals out of 338 fishes from the Slocum River estuary on Buzzards Bay, Massachusetts, USA;  ‘an adult with compacted and fused vertebrae’ from Chesapeake Bay, USA | Musick and Hoff (1968)  Kroger and Guthrie (1973) |
| Cyprinidae | | |
| Common carp, *Cyprinus carpio* L. 1758 | all individuals of Aischgrunder Karpfen, the German carp breed;  seven stumpbody individuals out of 671 fishes tank-reared in Saudi Arabia | Wunder (1949)  Al-Harbi (2001) |
| Catla, [*Gibelion*](http://researcharchive.calacademy.org/research/ichthyology/catalog/fishcatget.asp?genid=1749) [*catla*](http://researcharchive.calacademy.org/research/ichthyology/catalog/fishcatget.asp?spid=3130) [(Hamilton](http://en.wikipedia.org/wiki/Francis_Buchanan-Hamilton) [1822)](http://researcharchive.calacademy.org/research/ichthyology/catalog/getref.asp?id=2031) | the only hump-backed individual from the tank near Calcutta, India | Law (1944) |
| Yellowfin barbell, [*Luciobarbus*](http://researcharchive.calacademy.org/research/ichthyology/catalog/fishcatget.asp?genid=1737) [*xanthopterus*](http://researcharchive.calacademy.org/research/ichthyology/catalog/fishcatget.asp?spid=1962) [Heckel](http://en.wikipedia.org/wiki/Johann_Jakob_Heckel) [1843](http://researcharchive.calacademy.org/research/ichthyology/catalog/getref.asp?id=2066) | the only individual with 22 fused vertebrae from Mesopotamia, Iraq | Jawad *et al.* (2015) |
| Mesopotamian barb, *Mesopotamichthys sharpeyi* (Günther 1874) | an individual with 9 fused vertebrae from Mesopotamia, Iraq | Al-Hassan and Naama (1986) |
| Ictaluridae | | |
| Channel cafish, [*Ictalurus*](http://researcharchive.calacademy.org/research/ichthyology/catalog/fishcatget.asp?genid=1020) [*punctatus*](http://researcharchive.calacademy.org/research/ichthyology/catalog/fishcatget.asp?spid=54206) [(Rafinesque](http://en.wikipedia.org/wiki/Constantine_Samuel_Rafinesque) [1818)](http://researcharchive.calacademy.org/research/ichthyology/catalog/getref.asp?id=3586) | solitary individuals with ‘stumpbody morphology’ from the farmed stock | Dunhan *et al.* (1991) |
| Osmeridae | | |
| European smelt, [*Osmerus*](http://researcharchive.calacademy.org/research/ichthyology/catalog/fishcatget.asp?genid=7290) [*eperlanus*](http://researcharchive.calacademy.org/research/ichthyology/catalog/fishcatget.asp?spid=10807) L. 1758 | solitary individuals with ‘shortening of the spine’ from the Elbe estuary, Germany | Pohl (1990) |
| Plecoglossidae | | |
| Ayu s[weetfish](https://de.pons.com/%C3%BCbersetzung/englisch-deutsch/whitefish), [*Plecoglossus*](http://researcharchive.calacademy.org/research/ichthyology/catalog/fishcatget.asp?genid=6253) [*altivelis*](http://researcharchive.calacademy.org/research/ichthyology/catalog/fishcatget.asp?spid=10500) [(Temminck](http://en.wikipedia.org/wiki/Coenraad_Jacob_Temminck) & [Schlegel](http://en.wikipedia.org/wiki/Hermann_Schlegel) [1846)](http://researcharchive.calacademy.org/research/ichthyology/catalog/getref.asp?id=4374) | dumpy and hump-backed individuals constituting up to 0.6% in wild populations and up to 3% in hatchery-reared fish from Honshu waters, Japan | Komada (1980) |
| Salmonidae | | |
| [Lake](https://de.pons.com/%C3%BCbersetzung/englisch-deutsch/Lake) [Constance](https://de.pons.com/%C3%BCbersetzung/englisch-deutsch/Constance) [whitefish](https://de.pons.com/%C3%BCbersetzung/englisch-deutsch/whitefish)es, [*Coregonus*](http://researcharchive.calacademy.org/research/ichthyology/catalog/fishcatget.asp?genid=6253) [*macrophthalmus*](http://researcharchive.calacademy.org/research/ichthyology/catalog/fishcatget.asp?spid=10500) Nüsslin [1882](http://researcharchive.calacademy.org/research/ichthyology/catalog/getref.asp?id=14296) and [*C.*](http://researcharchive.calacademy.org/research/ichthyology/catalog/fishcatget.asp?genid=6253) [*wartmanni*](http://researcharchive.calacademy.org/research/ichthyology/catalog/fishcatget.asp?spid=28057) [(Bloch](http://en.wikipedia.org/wiki/Marcus_Elieser_Bloch) [1784)](http://researcharchive.calacademy.org/research/ichthyology/catalog/getref.asp?id=19645) | crimppled individuals constituting up to 6% of catches from [Lake](https://de.pons.com/%C3%BCbersetzung/englisch-deutsch/Lake) [Constance](https://de.pons.com/%C3%BCbersetzung/englisch-deutsch/Constance) at Bregenz, Austria | Wunder (1975*b*) |
| Maraena whitefish, [*Coregonus*](http://researcharchive.calacademy.org/research/ichthyology/catalog/fishcatget.asp?genid=6253) [*maraena*](http://researcharchive.calacademy.org/research/ichthyology/catalog/fishcatget.asp?spid=27993) [(Bloch](http://en.wikipedia.org/wiki/Marcus_Elieser_Bloch) [1779)](http://researcharchive.calacademy.org/research/ichthyology/catalog/getref.asp?id=16967) | the only hump-backed individual from Lake Wielkie Tuczno, Poland | Kulmatycki (1928) |
| Arctic char *Salvelinus alpinus* (L. 1758) | frequent deformity in the natural population from Transbaikalian Lake Dzhelo, Russia | Alekseyev (2016) |
| Atlantic salmon, *Salmo salar* L. 1758 | frequent deformity in farmed stocks, called the short-spined or short tail | Kvellestad *et al*. (2000),  Witten *et al*. (2005) |
| Brown trout***,*** [*Salmo*](http://researcharchive.calacademy.org/research/ichthyology/catalog/fishcatget.asp?genid=136) [*trutta*](http://researcharchive.calacademy.org/research/ichthyology/catalog/fishcatget.asp?spid=10789) L. 1758 | the only hump-backed individual from Scotland;  11 individuals exhibiting ‘typical cyprinid conformation’ out of 191 studied fish farmed in England | Ritchie (1908)  Poynton (1987) |
| Gadidae | | |
| Atlantic cod, *Gadus morhua* L. 1758 | solitary individuals with ‘deformed spine’ from British waters;  6-7 individuals ‘of humpbacked appearance’ from the Firth of Forth, Scotland;  deformed individuals constituting up to 20% of catches from the mouth of the River Elbe, Germany;  deformed individuals whose prevalence varied seasonally from 0.4 to 50% in the Elbe estuary;  deformed individuals constituting up to 13.5% of catches from the German Wadden Sea | Dyce (1860)  Smith (1867)  Wunder (1971)  Möller (1983, 1984)  Hilger (1992) |
| Haddock, [*Melanogrammus*](http://researcharchive.calacademy.org/research/ichthyology/catalog/fishcatget.asp?genid=3044) [*aeglefinus*](http://researcharchive.calacademy.org/research/ichthyology/catalog/fishcatget.asp?spid=12355) L. 1758 | solitary individuals with ‘deformed spine’ from British waters.  Two individuals with 29 and 15 deformed vertebrae from Masfjorden, Western Norway | Dyce (1860)  Jawad *et al*. (2018) |
| Haddock, [*Melanogrammus*](http://researcharchive.calacademy.org/research/ichthyology/catalog/fishcatget.asp?genid=3044) [*aeglefinus*](http://researcharchive.calacademy.org/research/ichthyology/catalog/fishcatget.asp?spid=12355) L. 1758 | solitary individuals with ‘deformed spine’ from British waters | Dyce (1860) |
| Fundulidae | | |
| Banded topminnow, [*Fundulus*](http://researcharchive.calacademy.org/research/ichthyology/catalog/fishcatget.asp?genid=535) [*cingulatus*](http://researcharchive.calacademy.org/research/ichthyology/catalog/fishcatget.asp?spid=13704) [Valenciennes](http://en.wikipedia.org/wiki/Achille_Valenciennes) [1846](http://researcharchive.calacademy.org/research/ichthyology/catalog/getref.asp?id=1011) | *stubby* phenotype in the laboratory reared strain with complex genetic determination | Thomerson (1966) |
| Poecilidae | | |
| Guppy, [*Poecilia*](http://researcharchive.calacademy.org/research/ichthyology/catalog/fishcatget.asp?genid=440) [*reticulata*](http://researcharchive.calacademy.org/research/ichthyology/catalog/fishcatget.asp?spid=58598) [Peters](http://sv.wikipedia.org/wiki/Wilhelm_Peters) [1859](http://researcharchive.calacademy.org/research/ichthyology/catalog/getref.asp?id=18542) | *palla* mutant in the laboratory-reared strain | Lodi (1978) |
| Blackstripe livebearer, [*Poeciliopsis*](http://researcharchive.calacademy.org/research/ichthyology/catalog/fishcatget.asp?genid=7676) [*prolifica*](http://researcharchive.calacademy.org/research/ichthyology/catalog/fishcatget.asp?spid=24956) Miller [1960](http://researcharchive.calacademy.org/research/ichthyology/catalog/getref.asp?id=10418) | *stubby* mutant in the laboratory-reared strain | Schultz (1963) |
| Adrianichthyidae |  |  |
| Japanese rice fish, [*Oryzias*](http://researcharchive.calacademy.org/research/ichthyology/catalog/fishcatget.asp?genid=5355) [*latipes*](http://researcharchive.calacademy.org/research/ichthyology/catalog/fishcatget.asp?spid=13553) [(Temminck](http://en.wikipedia.org/wiki/Coenraad_Jacob_Temminck) & [Schlegel](http://en.wikipedia.org/wiki/Hermann_Schlegel) [1846)](http://researcharchive.calacademy.org/research/ichthyology/catalog/getref.asp?id=4374) | *fused* mutant in the laboratory-reared strain and "wild-fused" phenotype from a wild population from outskirts of Nagoya, Japan | Aida (1930), Yamamoto *et al*. (1963), Ogawa (1965) |
| Sciaenidae |  |  |
| Spot croaker, [*Leiostomus*](http://researcharchive.calacademy.org/research/ichthyology/catalog/fishcatget.asp?genid=518) [*xanthurus*](http://researcharchive.calacademy.org/research/ichthyology/catalog/fishcatget.asp?spid=19657) [Lacepède](http://en.wikipedia.org/wiki/Bernard_Germain_de_Lac%C3%A9p%C3%A8de) [1802](http://researcharchive.calacademy.org/research/ichthyology/catalog/getref.asp?id=4929) | the only individual ‘with compacted and fused vertebrae’ from the Core Sound, North Carolina, USA | Kroger and Guthrie (1973) |
| Mullidae |  |  |
| Red mullet, [*Mullus*](http://researcharchive.calacademy.org/research/ichthyology/catalog/fishcatget.asp?genid=143) [*barbatus*](http://researcharchive.calacademy.org/research/ichthyology/catalog/fishcatget.asp?spid=19548) L. 1758 | the only hump-backed individual from Izmir Bay, Aegean Sea, Turkey | Jawad and Akyol (2018) |
| Mugilidae |  |  |
| Flathead grey mullet, [*Mugil*](http://researcharchive.calacademy.org/research/ichthyology/catalog/fishcatget.asp?genid=152) [*cephalus*](http://researcharchive.calacademy.org/research/ichthyology/catalog/fishcatget.asp?spid=19549) [L](http://en.wikipedia.org/wiki/Carl_Linnaeus)[. 1758](http://researcharchive.calacademy.org/research/ichthyology/catalog/getref.asp?id=2787) | an individual with 6 fused vertebrae from Benghazi area, Libya | Jawad (2002) |
| Cichlidae |  |  |
| Blue tilapia, *Oreochromis aureus* [(Steindachner](http://en.wikipedia.org/wiki/Franz_Steindachner) [1864)](http://researcharchive.calacademy.org/research/ichthyology/catalog/getref.asp?id=13938), Nile tilapia *O. niloticus* (L. 1758), “Florida red tilapia,” (a strain derived from *O*. *urolepis hornorum* ♀ × *O*. *mossambica* ♂ matings) | solitary stumpbody individuals in farmed and laboratory stocks | Tave *et al.* (1982, 2011), Eissa *et al.* (2009) |

REFERENCES

Aida, T. (1930). Further genetical studies of *Aplocheilus latipes*. *Genetics, 15*, 1–16.

Alekseyev, S.S. 2016. Distribution, diversity and diversification of the Arctic char *Salvelinus alpinus* (L.) complex (Salmoniformes, Salmonidae) in Siberia. Doctoral Dissertation. Moscow, Russia: Moscow State University, Faculty of Biology [in Russian].

Al-Harbi, A. H. (2001). Skeletal deformities in cultured common carp *Cyprinus carpio* L. *Asian Fisheries Science, 14*, 247–254.

Al-Hassan L. A. J., & Naama, A. K. (1986). More vertebral anomalies in ﬁshes from Euphrates River, Iraq. *Indian Journal of Fisheries, 33*, 237–241.

Dunhan, R.A., Smitherman, R.O., & Bondari, K. (1991). Lack of inheritance of stumpbody and taillessness in channel catfish. *Progressive Fish-Culturist, 53*, 101–105.

Dyce, R. (1860). On the identity of *Morrhua punctata* and *Morrhua vulgaris. Annals and Magazine of Natural History, 5*, 366–369.

Eissa, A.E., Moustafa, M., El-Husseiny, I.N., Saeid, S., Saleh, O., & Borhan, T. (2009). Identification of some skeletal deformities in freshwater teleost raised in Egyptian aquaculture. *Chemosphere, 77*, 419–425.

Hilger, I. (1992). Spinal compression of Atlantic cod *Gadus morhua* from the German Wadden Sea. *Diseases of Aquatic Organisms, 13*, 83–88.

Jawad, L. A. (2002). A record of fish anomalies from Benghazi area, Libya. *Teratology, 65*, 1–2.

Jawad L. A., & Akyol O. (2018). Vertebral anomalies in *Mullus barbatus* (Actinopterygiidae: Osteichthyes: Mullidae), collected from Izmir Bay, North-eastern Aegean Sea, Turkey. *International Journal of Marine Science, 8*, 59–65.

Jawad, L. A., Al-Faisal, A. J., & Al-Mukhtar, M. (2015). A case of vertebral coalescence in *Luciobarbus xanthopterus* (Heckel, 1843) (Pisces: Cyprinidae) obtained from the lower reaches of Mesopotamia. *Travaux du Muséum National d’Histoire Naturelle «Grigore Antipa», 57*, 127–132.

Jawad, L. A., Hansen, T. J., Meier, S., Skjaeraasen, J. E., & Fjelldal, P. G. (2018). Vertebral column deformity in adult wild haddock (*Melanogrammus aeglefinus*). *Journal of Fish Diseases.* doi:10.1111/jfd.12903

Kulmatycki, W. (1928). O anomalji w budowie kręgosłupa sieji szlachetnej. *Kosmos 53, Seria A,* 147–150.

Komada, N. (1980). Incidence of gross malformations and vertebral anomalies of natural and hatchery *Plecoglossus altivelis. Copeia, 1980*, 29–35.

Kroger, R. L., & Guthrie, J. F. (1973). Additional anomalous menhaden and other fishes. *Chesapeake Science, 14*, 112–116.

Kvellestad, A., Høie, S., Thorud, K., Tørud, B., & Lyngøy, A. (2000). Plathyspondyly and shortness of vertebral column in farmed Atlantic salmon *Salmo salar* in Norway—description and interpretation of pathologic changes. *Diseases of Aquatic Organisms, 39*, 97–108.

Law, N.C. (1944). A hump backed carp, *Catla catla* (Hamilton). *Proceedings of the National Institute of Sciences of India, 10*, 97–103.

Lodi, E. (1978). Palla, A hereditary vertebral deformity in the guppy, *Poecilia*

*reticulata* Peters, Pisces, Osteichthyes. *Genetica, 48*, 197–200.

Möller, H. (1983). High skeletal deformation rates of cod in the Elbe estuary. *Bulletin of the European Association of Fish* Pathologists, 3, 7–8.

Möller, H. (1984). Dynamics of fish diseases in the lower Elbe River. *Helgoländer wissenschaftlinche Meeresuntersuchungen, 37*, 389–413.

Musick, J. A., & Hoff, J. G. (1968). Vertebral anomalies in humpbacked specimens of menhaden, *Brevoortia tyrannus*. *Transactions of the American Fisheries Society,* 97, 277–278.

Ogawa, N.M. (1965). A phylogenetic study of the vertebral fused (f) in the medaka, *Oryzias latipes*. *Embryologia, 9*, 13–33.

Pohl, C. (1990). Skeletal deformities and trace metal contents of European smelt, *Osmerus eperlanus*, in the Elbe estuary. *Meeresforschung, 33*, 76–89.

Poynton, S. (1987). Vertebral column abnormalities in brown trout, Salmo trutta L. Journal of Fish Diseases, 10, 53–57.

Ritchie, J. (1908). A hump-backed trout from Stranraer. *Annals of Scottish Natural History, No. 68*, 223–227.

Schultz, R.J. (1963). Stubby, a hereditary vertebral deformity in the viviparous fish *Poeciliopsis prolifica*. Copeia, 2, 325–330.

Smith, J. A. (1867). Notice of various specimens of the deformed variety of *Morrhua vulgaris*, the common codfish, the 'Lord Fish' of Yarrel, recently taken in the Firth of Forth. *Proceedings of the Royal Physical Society of Edinburgh, 3*, 302-303.

Tave, D., Bartels, J.E., & Smitherman, R.O. (1982). Stumpbody *Sarotherodon aureus* (Steindachner) (*Tilapia aurea*) and tail-less *S. niloticus* (L.) (*T. nilotica*): two vertebral anomalies and their effects on body length. *Journal of Fish Diseases, 5*, 487–494.

Tave, D., Jo, J.-Y., & Kim, D.S. (2011). Gross abnormalities in tilapia. *Fisheries and Aquatic Science, 14*, 148–160.

Thomerson, J. E. (1966). Tbe stubby vertebral deformity in aquarium-raised *Fundulus cingulatus*. *Copeia, 1966*, 878–879.

Witten, P.E., Gil Martens, L., Hall, B.K., Huysseune, A., Obach, A. (2005). Compressed vertebrae in Atlantic salmon (*Salmo salar*): evidence for metaplastic chondrogenesis as a skeletogenic response late in ontogeny. *Diseases of Aquatic Organisms, 64*, 237–246.

Wunder, W. (1949). Wirbelsäulenverkürzung als rassenbildendes Merkmal beim Aischgründer Karpfen. *Wilhelm Roux' Archiv Entwicklungsmechanik, 144*, 1–24.

Wunder, W. (1968). Hochrückigkeit beim Aal (*Anguilla anguilla* L.) bedingt durch Wirbelsäulenverkürzung. *Biologisches Zentralblatt, 87*, 325–331.

Wunder, W. (1971). Missbildungen beim Kabeljau (*Gadus morhua*) verursacht durch Wirbelsaulenverkiirzung. *Helgoländer wissenschaftlinche Meeresuntersuchungen, 22*, 201–212.

Wunder, W. (1975*b*). Verkriippelte Felchen aus den Bodensee. Blaufelchen (*Coregonus wartrnanni* Bloch) und Gangfisch (*Coregonus macrophthalmus* L.). Ursache: Wirbelsaulenverkiirzung (Toxische Osteosklerose). *Zoologischer Anzeiger, 194*, 279-292.

Yamamoto, T., Tomita, H., & Matsuda, N. (1963). Hereditary and nonheritable vertebral anchylosis in the medaka, *Oryzias latipes*. *The* Japanese Journal of Genetics*, 38*, 36–47.

**S1 Fig.** Photographs of two habitats at the main sampling (no. 1) on the middle Genale River – (A) the continuous pool, and (B) upper part of rapids downstream of the pool.


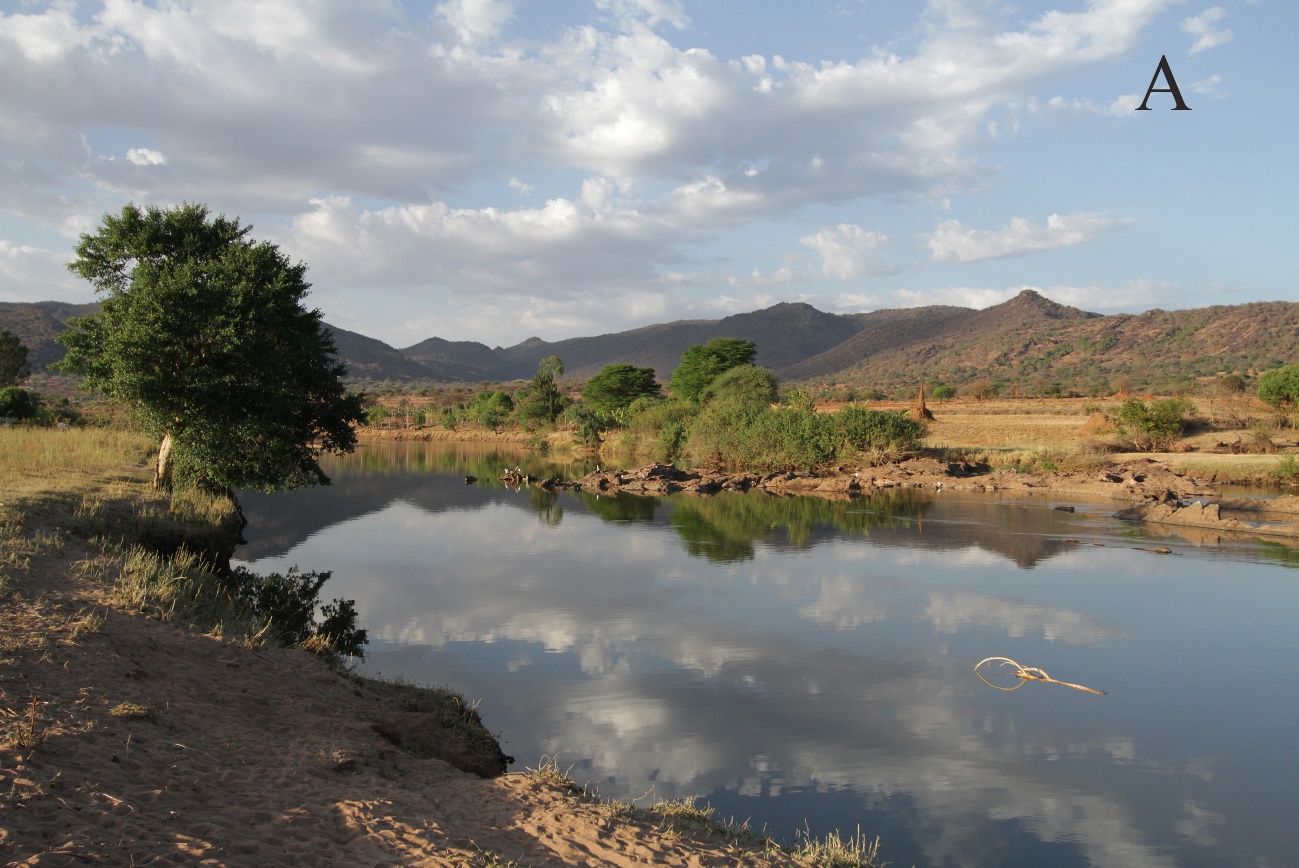


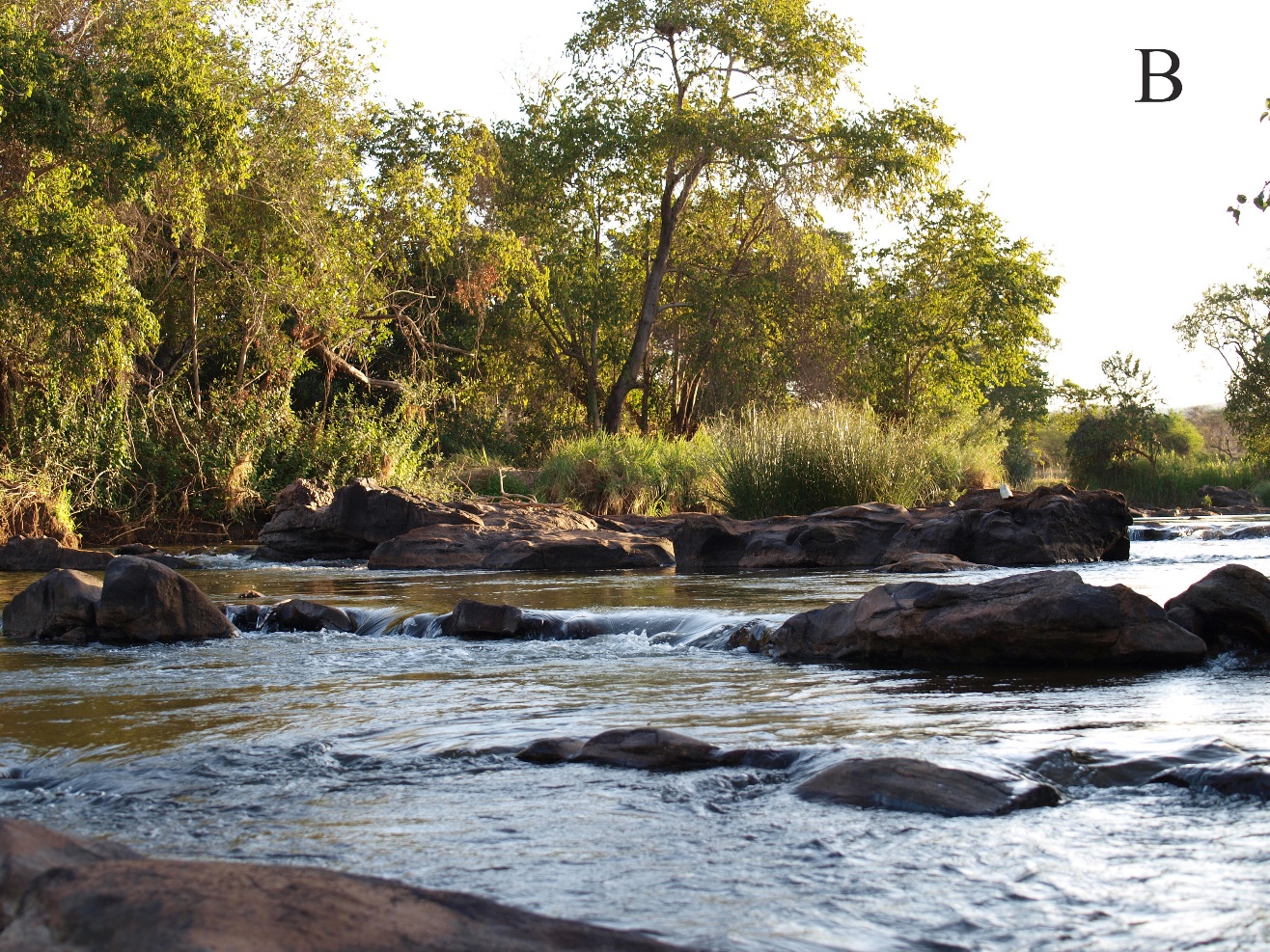


**S2 Table.** Size characteristics of the cast and gill net catches for different *Labeobarbus* forms from the middle Genale assemblage sampled in 2009 and 2019 and size characteristics of the aged individuals from the gill net catches of 2019

| **Form/sample/**  **age (years)** | **N** | **Standard length, mm** | | | **Head length, mm** | | |
| --- | --- | --- | --- | --- | --- | --- | --- |
|  |  | **min** | **median** | **max** | **min** | **median** | **max** |
| **Short (SH)** | | | | | | | |
| Gill net sample of 2019 | 11 | 177 | 201 | 244 | 50.7 | 58.2 | 65 |
| Cast net sample of 2009 | 0 | - | - | - | - | - | - |
| Gill net sample of 2009 | 35 | 112 | 201 | 291 | 34 | 52 | 74 |
| Aged sample of 2009 | 19 | 112 | 212 | 291 | 34 | 56 | 74 |
| 1 | 2 | - | 112 | - | - | 34 | - |
| 2 | 3 | 136 | 140 | 188 | 38.5 | 39 | 51 |
| 3 | 6 | 158 | 182 | 228 | 44 | 51 | 62 |
| 4 | 4 | 212 | 227 | 232 | 56 | 59.5 | 61 |
| 5 | 5 | 218 | 226 | 239 | 58 | 60 | 63 |
| 6 | 3 | 258 | 276 | 291 | 60 | 62 | 72 |
| **Lipped short (LP SH)** | | | | | | | |
| Cast net sample of 2009 | 0 | - | - | - | - | - | - |
| Gill net sample of 2009 | 6 | 107 | 200,5 | 277 | 28 | 54 | 68 |
| Aged sample of 2009 | 2 | 107 | 192 | 277 | 28 | 48 | 68 |
| 1 | 1 | - | 107 | - | - | 28 | - |
| 4 | 1 | - | 277 | - | - | 68 | - |
| **Generalized (GN)** | | | | | | | |
| Gill net sample of 2019 | 7 | 171 | 213 | 237 | 43.4 | 50.7 | 63.3 |
| Cast net sample of 2009 | 20 | 51 | 95 | 114 | - | - | - |
| Gill net sample of 2009 | 41 | 81 | 195 | 375 | 20 | 46 | 90 |
| Aged sample of 2009 | 24 | 81 | 191 | 375 | 20 | 44.5 | 90 |
| 1 | 3 | 81 | 81 | 100 | 20 | 22 | 26 |
| 2 | 6 | 105 | 144 | 155 | 25.5 | 35 | 38,5 |
| 3 | 4 | 178 | 181 | 201 | 41 | 44 | 45 |
| 4 | 4 | 199 | 215 | 221 | 47 | 49.5 | 52 |
| 5 | 2 | 245 | 260.5 | 276 | 58 | 60.5 | 63 |
| 6 | 3 | 283 | 339 | 356 | 67 | 78.5 | 85 |
| 7 | 1 | - | 356 | - | - | 84 | - |
| 8 | 1 | - | 375 | - | - | 90 | - |
| **Lipped (LP)** | | | | | | | |
| Gill net sample of 2019 | 4 | 158 | 220.5 | 298 | 41.7 | 62.15 | 78.6 |
| Cast net sample of 2009 | 1 | - | 107 | - | - | - | - |
| Gill net sample of 2009 | 21 | 106 | 173 | 459 | 28 | 43.4 | 129 |
| Aged sample of 2009 | 12 | 106 | 203 | 459 | 28 | 55 | 129 |
| 1 | 2 | - | 106 | - | 27.5 | 28.75 | 30 |
| 2 | 2 | 112 | 135 | 158 | 30 | 35 | 38,5 |
| 3 | 2 | 172 | 173.5 | 175 | 42 | 45 | 48 |
| 4 | 2 | 231 | 232 | 233 | - | 62 | - |
| 5 | 1 | - | 269 | - | - | 71 | - |
| 6 | 1 | - | 280 | - | - | 69 | - |
| 9 | 1 | - | 363 | - | - | 85 | - |
| 12 | 1 | - | 459 | - | - | 129 | - |
| **Jubae (JB)** | | | | | | | |
| Gill net sample of 2019 | 5 | 139 | 174 | 216 | 31.1 | 38.2 | 49.7 |
| Cast net sample of 2009 | 32 | 74.5 | 83.65 | 111.2 | - | - | - |
| Gill net sample of 2009 | 36 | 89 | 180.5 | 349 | 20 | 37.5 | 73 |
| Aged sample of 2009 | 26 | 89 | 206 | 349 | 20 | 44.5 | 73 |
| 1 | 3 | 89 | 93 | 109 | 20 | 21.5 | 24 |
| 2 | 5 | 113 | 133 | 146 | 20.5 | 30 | 34 |
| 3 | 6 | 159 | 180.5 | 222 | 35 | 37.5 | 51 |
| 4 | 5 | 232 | 237 | 260 | 47 | 50 | 51 |
| 5 | 2 | 292 | 293 | 294 | 59 | 59.5 | 60 |
| 6 | 2 | 283 | 296.5 | 310 | 57 | 59 | 61 |
| 7 | 1 | - | 325 | - | - | 64 | - |
| 8 | 1 | - | 349 | - | - | 73 | - |
| 9 | 1 | - | 340 | - | - | 70 | - |
| **Smiling (SM)** | | | | | | | |
| Gill net sample of 2019 | 8 | 142 | 204.5 | 260 | 35.2 | 50.55 | 64.1 |
| Cast net sample of 2009 | 5 | 84.2 | 115 | 150 | - | - | - |
| Gill net sample of 2009 | 24 | 82 | 150 | 360 | 20.5 | 37.5 | 82 |
| Aged sample of 2009 | 15 | 82 | 134 | 360 | 20.5 | 33 | 82 |
| 1 | 5 | 82 | 88 | 146 | 20.5 | 22 | 35 |
| 2 | 4 | 87 | 111.5 | 134 | 21 | 26.75 | 33 |
| 4 | 3 | 157 | 182 | 183 | 40 | 43.5 | 46 |
| 5 | 2 | 244 | 248 | 252 | 60 | 63 | 66 |
| 7 | 1 | - | 360 | - | - | 82 | - |
| **Hybrid (HB)** | | | | | | | |
| Gill net sample of 2019 | 5 | 107 | 167 | 247 | 28.3 | 43.6 | 57.4 |
| Cast net sample of 2009 | 10 | 76 | 82.5 | 148 | - | - | - |
| Gill net sample of 2009 | 19 | 84 | 204 | 296 | 21 | 45 | 66 |
| Aged sample of 2009 | 6 | 106 | 202.5 | 296 | 21 | 43.75 | 66 |
| 2 | 1 | - | 106 | - | - | 21 | - |
| 3 | 2 | 176 | 188.5 | 201 | 39 | 40.75 | 42.5 |
| 4 | 1 | - | 204 | - | - | 45 | - |
| 5 | 1 | - | 256 | - | - | 58 | - |
| 6 | 1 | - | 296 | - | - | 66 | - |
| **Piscivorous (PS)** | | | | | | | |
| Gill net sample of 2019 | 5 | 161 | 256 | 274 | 48 | 80.7 | 85.9 |
| Cast net sample of 2009 | 1 | - | 127 | - | - | - | - |
| Gill net sample of 2009 | 39 | 106 | 243 | 426 | 31 | 72 | 128 |
| Aged sample of 2009 | 28 | 106 | 245 | 426 | 31 | 73 | 128 |
| 1 | 1 | - | 106 | - | - | 31 | - |
| 2 | 2 | 112 | 114.5 | 117 | - | 33 | - |
| 3 | 5 | 172 | 183 | 196 | 49 | 53.5 | 56 |
| 4 | 3 | 212 | 229 | 266 | 62 | 65 | 74 |
| 5 | 7 | 202 | 246 | 279 | 55 | 74 | 90 |
| 6 | 6 | 244 | 304.5 | 322 | 68 | 87 | 96 |
| 7 | 1 | - | 329 | - | - | 97 | - |
| 9 | 1 | - | 393 | - | - | 126 | - |
| 12 | 1 | - | 400 | - | - | 113 | - |
| 14 | 1 | - | 426 | - | - | 128 | - |

**S5 Table**. Counts and morphometrics (Provided as separated XLSL-file).

**S3 Table.** Sample size of subsets analyzed in study.

| Subsets | Generalized | Short | Lipped | Jubae | Smiling | Piscivorous | Statistical method |
| --- | --- | --- | --- | --- | --- | --- | --- |
| Vertebral column statistics (X-Ray + dry skeletons) | 78 | 52 | 26 | 78 | 48 | 46 | Basal descriptive statistics, KW* test |
| Correlation between deformed vertebrae and body height in Short (Fig. 3) | - | 51 | - | - | - | - | Pearson correlation |
| External measurements PCA/CVA (Fig. 4) | 23 | 28 | - | - | - | - | PCA, CVA |
| External measurements for single characters 2009 (Fig. 5) | 41 | 35 | 17 | - | - | - | KW test |
| External measurements for single characters (Fig. 6) | 2009 = 41  2019 = 7 | 2009 = 35  2019 = 11 | - | - | - | - | Mann-Whitney U test |
| Distribution of deformed vertebrae along vert. column (Fig. 7 and S10) | 78 | 52 | 26 | 78 | 48 | 46 | Fisher’s exact test |
| Age analysis (total) | 24 | 23 | 12 | 26 | 15 | 28 |  |
| Age analysis by  year classes (Fig. 8) | 1y = 3  2y = 6  3y = 4  4y = 4  5y = 2  6y = 3  7y = 1  8y = 1 | 1y = 2  2y = 3  3y = 6  4y = 4  5y = 5  6y = 3 | 1y = 2  2y = 2  3y = 2  4y = 2  5y = 1  6y = 1  9y = 1  12y = 1 | 1y = 3  2y = 5  3y = 6  4y = 5  5y = 2  6y = 2  7y = 1  8y = 1  9y = 1 | 1y = 5  2y = 4  4y = 3  5y = 2  7y = 1 | 1y = 1  2y = 2  3y = 5  4y = 3  5y = 7  6y = 6  7y = 1  9y = 1  12y = 1  14y = 1 | Mann-Whitney U test |
| Gonads | 40 | 30 | 6 | - | - | - |  |

*KW = Kruskal-Wallis;

**S1 Supporting material.** Data on effect size of samples for Kruskal-Wallis test (A) and Mann-Whitney U test (B.)

1. Effect size of samples for Kruskal-Wallis tests (to Figure 5).

(The interpretation of Eta^2^ values commonly in published litterature are: 0.01- < 0.06 (small effect), 0.06 - < 0.14 (moderate effect) and >= 0.14 (large effect). **Eta^2^ values are in bold**.

> library(rstatix)

> kruskal_effsize(data=Kruskall_Wallis_Short_NEC_LIP_SL_HL_H_cpd_h,

+ formula = HL_SL ~ Morph, ci = FALSE, conf.level = 0.95, ci.type = "perc", nboot = 1000)

# A tibble: 1 x 5

.y. n effsize method magnitude

* *<chr>* *<int>* *<dbl>* *<chr>* *<ord>*

1 HL_SL 98 **0.584** eta2[H] large

> kruskal_effsize(data=Kruskall_Wallis_Short_NEC_LIP_SL_HL_H_cpd_h,

+ formula = H_SL ~ Morph, ci = FALSE, conf.level = 0.95, ci.type = "perc", nboot = 1000)

# A tibble: 1 x 5

.y. n effsize method magnitude

* *<chr>* *<int>* *<dbl>* *<chr>* *<ord>*

1 H_SL 98 **0.581** eta2[H] large

> kruskal_effsize(data=Kruskall_Wallis_Short_NEC_LIP_SL_HL_H_cpd_h,

+ formula = h_SL ~ Morph, ci = FALSE, conf.level = 0.95, ci.type = "perc", nboot = 1000)

# A tibble: 1 x 5

.y. n effsize method magnitude

* *<chr>* *<int>* *<dbl>* *<chr>* *<ord>*

1. h_SL 98 **0.576** eta2[H] large
2. Table. Statistics of Mann-Whitney U-tests (two sample tests) to Figures 6 and 8.

| Comparisons | z | p-value | r (effect size)* |
| --- | --- | --- | --- |
| To Figure 6 | | | |
| HL/SL: Generalized 2009 vs 2019 | -3.0255 | 0.001 | 0.436 |
| HL/SL: Short 2009 vs 2019 | -2.2555 | 0.023 | 0.333 |
| H/SL: Generalized 2009 vs 2019 | -2.0313 | 0.040 | 0.293 |
| H/SL: Short 2009 vs 2019 | -0.6182 | 0.546 | 0.09 |
| h/SL: Generalized 2009 vs 2019 | -2.049 | 0.039 | 0.296 |
| h/SL: Short 2009 vs 2019 | -1.3143 | 0.194 | 0.194 |

* Effect size is considered small at r = 0.10, middle at r = 0.30, and large at r =>0.50.

**S2 Fig.** Two piscivorous barbs (sampled from the middle Genale *Labeobarbus* assemblage in 2009): A, normal individual (juvenile female, SL 277 mm) without modified vertebrae; B, short individual (female at II stage of maturity, SL 279 mm) with eight greatly modified vertebrae; C, modified segment of vertebral column of the latter individual. Some proportions of individuals A and B, respectively: head length/SL 0.285 *vs* 0.323; dorsal spine length/SL 0.162 *vs* 0.197; maximum body heigth/SL 0.245 *vs* 0.287; minimum body heigth/SL 0.108 *vs* 0.125; caudal peduncle length/SL 0.184 *vs* 0.197.

A

B


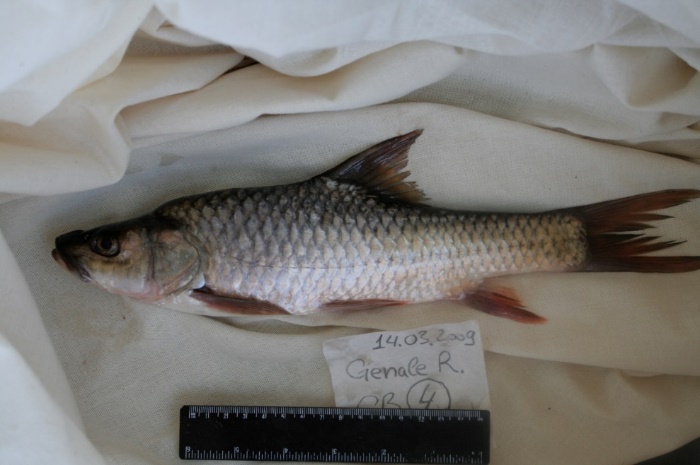

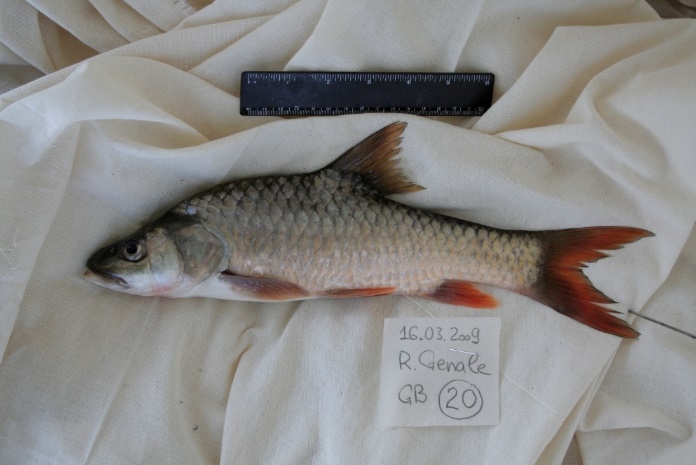


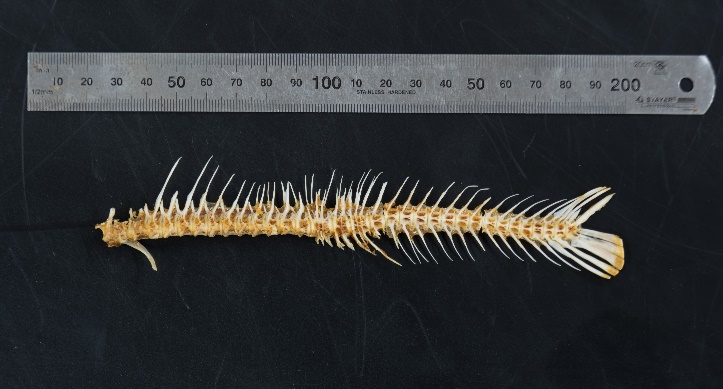


C

**S4 Table**. Gill net catch composition and percentage of different *Labeobarbus* morphs in the middle Genale assemblage in 2009 and 2019

| **Taxon/form** | **2009** | **2019** |
| --- | --- | --- |
| *Labeobarbus* | 400 | 179 |
| Shorts (SH) | 41 (10%) | 20 (11%) |
| Generalized (GN) | 217 (54%) | 103 (58%) |
| Lip (LP) | 24 (6%) | 10 (6%) |
| Jubae (JB) | 62 (16%) | 25 (14%) |
| Smiling (SM) | 9 (2%) | 9 (5%) |
| Hybrid (HY) | 14 (4%) | 6 (3%) |
| Pisciv (PS) | 33 (8%) | 6 (3%) |
| *Labeo* cf. *cylindricus* | 45 | 39 |
| *Mormyrus kannume* | 205 | 121 |
| *Bagrus urostigma* | 16 | 5 |

**S5 Table.** Eigenvectors of 10 most loaded characters to Fig. 4

| Character | PC1 | Character | PC2 |
| --- | --- | --- | --- |
| *VA* | -0.354 | *H* | -0.354 |
| *PV* | -0.309 | *lP* | 0.342 |
| *PrD* | -0.303 | *DL* | 0.336 |
| *lD* | -0.277 | *O* | 0.319 |
| *CH* | -0.254 | *h* | -0.294 |
| *PO* | -0.252 | *PD* | 0.253 |
| *h* | -0.233 | *R* | -0.246 |
| *lV* | -0.228 | *cpd* | 0.222 |
| *PD* | -0.223 | *IO* | -0.212 |
| *hA* | -0.221 | *PrD* | 0.211 |

**S6 Table.** Descriptive statistics of vertebral counts. SL – standard length, Vt – total number of vertebrae, Va – abdominal vertebrae, Vc – vertebrae of caudal region, Vi – vertebrae of intermediate region, Vc+i – caudal region vertebrae + intermediate vertebrae, Vpd – predorsal vertebrae. Range is given above, and average ± standard deviation (SD) below horizontal line.

| Morph | n | SL, mm | Vt | Va | Vc | Vi | Vc+i | Vpd |
| --- | --- | --- | --- | --- | --- | --- | --- | --- |
| GN | 82 | 51-375 | 40-43 | 17-20 | 16-19 | 4-6 | 21-24 | 10-12 |
|  |  | 168±73.9 | 41.43±0.61 | 18.54±0.67 | 17.78±0.70 | 5.11±0.61 | 22.89±0.77 | 10.70±0.49 |
| SH | 52 | 107-291 | 40-43 | 17-21 | 17-21 | 3-6 | 20-24 | 10-12 |
|  |  | 196±59.0 | 41.31±0.76 | 18.56±0.86 | 17.60±0.75 | 5.15±0.67 | 22.75±0.81 | 10.63±0.53 |
| LP | 22 | 106-363 | 40-42 | 17-22 | 16-22 | 4-6 | 21-24 | 10-11 |
|  |  | 194±70.26 | 41.32±0.65 | 18.27±0.55 | 17.64±0.85 | 5.41±0.59 | 23.05±0.72 | 10.82±0.39 |
| JB | 78 | 75-349 | 40-43 | 16-21 | 17-19 | 3-7 | 21-24 | 9-11 |
|  |  | 153±80.5 | 41.67±0.53 | 18.78±0.66 | 18.13±0.52 | 4.74±0.63 | 22.87±0.63 | 10.06±0.29 |
| SM | 42 | 82-360 | 40-42 | 17-18 | 18-20 | 3-6 | 23-25 | 10-11 |
|  |  | 172±68.8 | 41.10±0.48 | 17.48±0.51 | 18.81±0.59 | 4.81±0.67 | 23.62±0.58 | 10.21±0.42 |
| PS | 46 | 106-426 | 41-43 | 17-19 | 17-20 | 5-6 | 22-24 | 10-12 |
|  |  | 231±71.5 | 41.87±0.50 | 18.17±0.53 | 18.33±0.67 | 5.37±0.49 | 23.70±0.66 | 10.80±0.45 |

**S3 Fig.** An addition to Fig. 7. Distribution of deformed vertebrae along vertebral column in short morph (A and B), and other Genale barbs (A1 and B1) for individuals with 40 and 42 total vertebrae; the deformities were not analyzed in first four vertebrae (Weberian apparatus) as well as in the last (preural 1) vertebra for technical reasons.


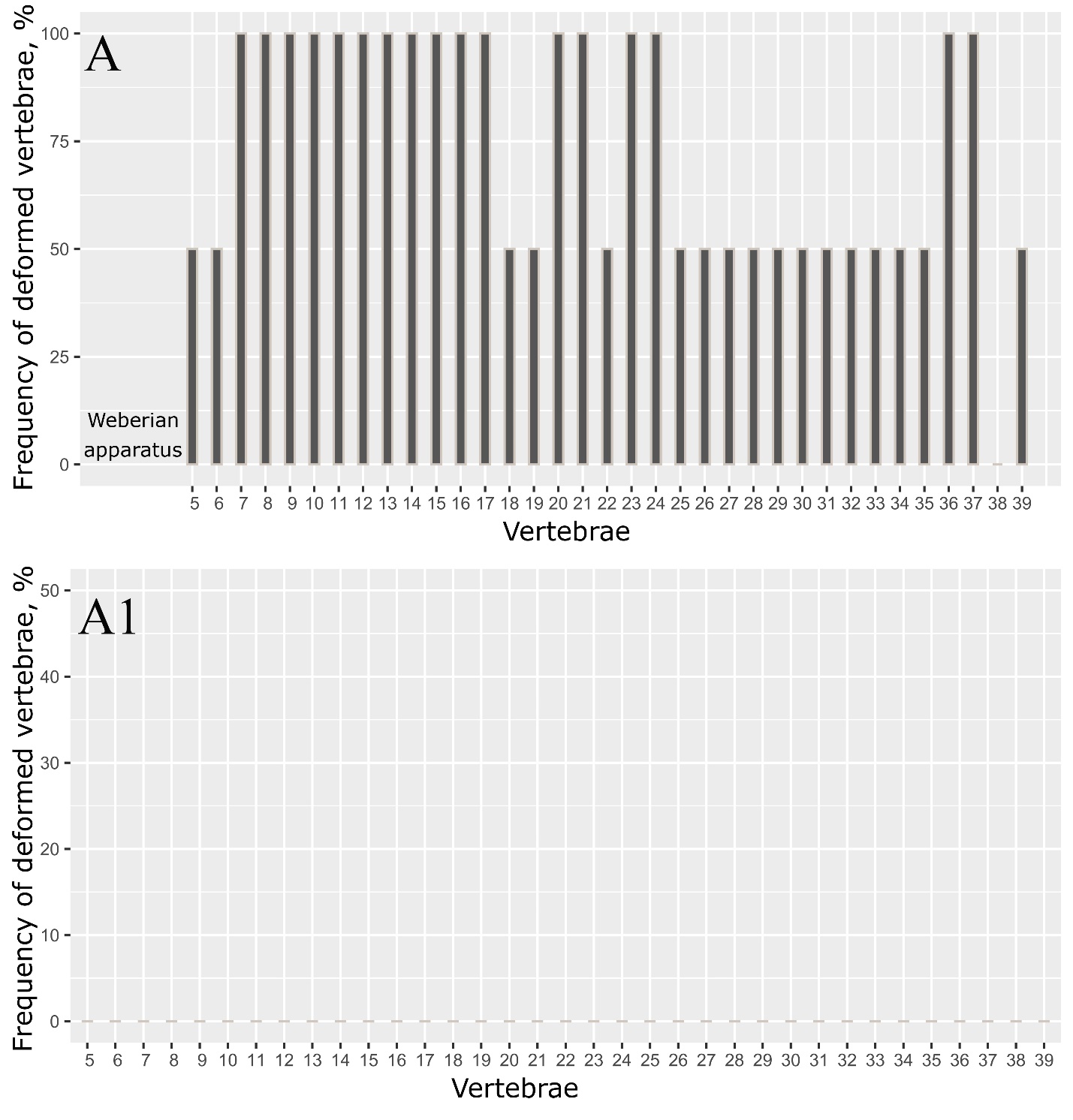


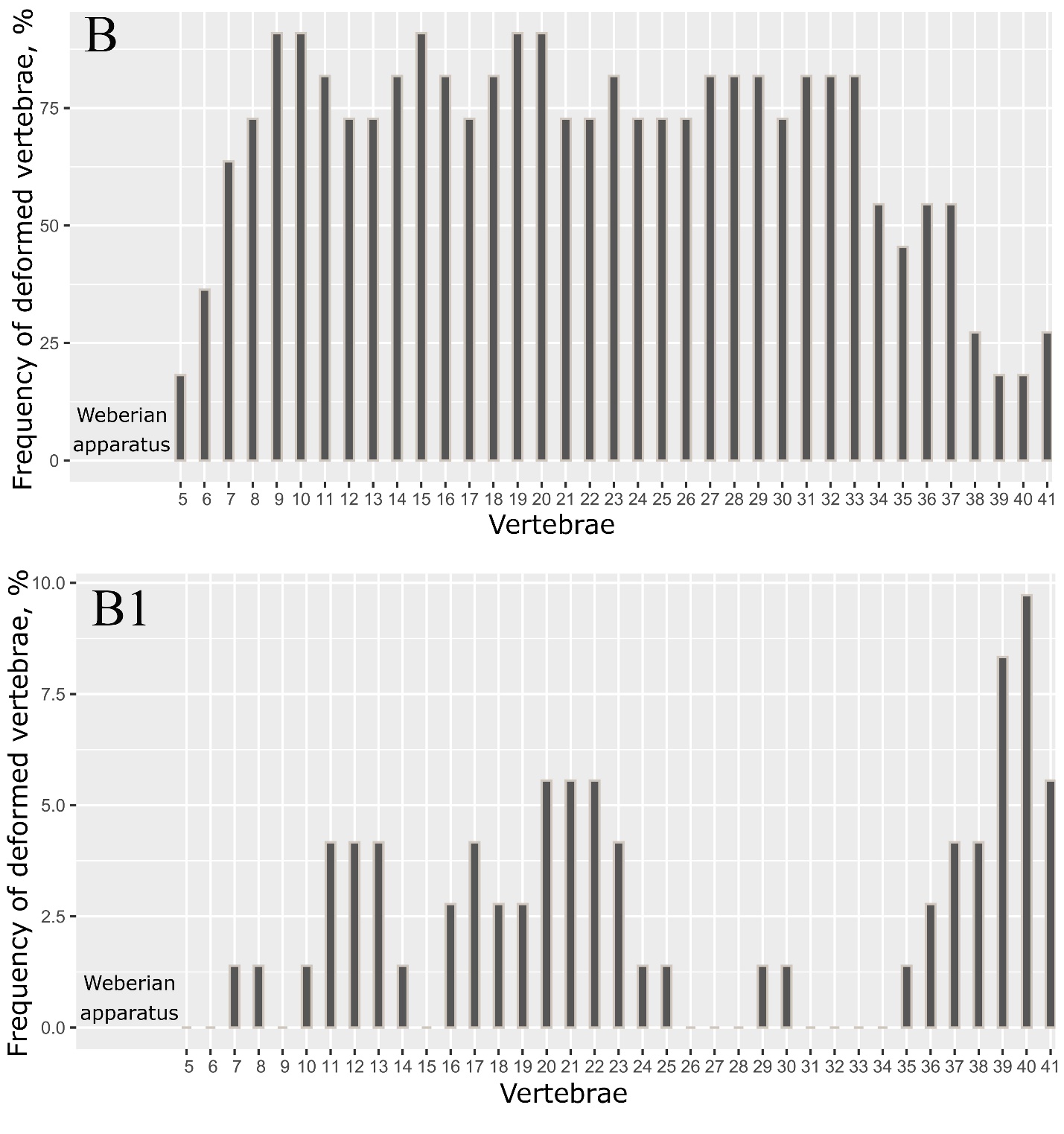


**S4 Fig.** Growth of short (SH), generalized (GN) and lipped (LP) forms in the middle Genale *Labeobarbus* assemblage in sample of 2009 estimated with standard length (SL). Median is shown as the horizontal black line inside the box. The box represents 1st and 3rd quartiles of variation. No significant differences between short and other morphs were detected within year classes 1-4 (Dunn’s test).


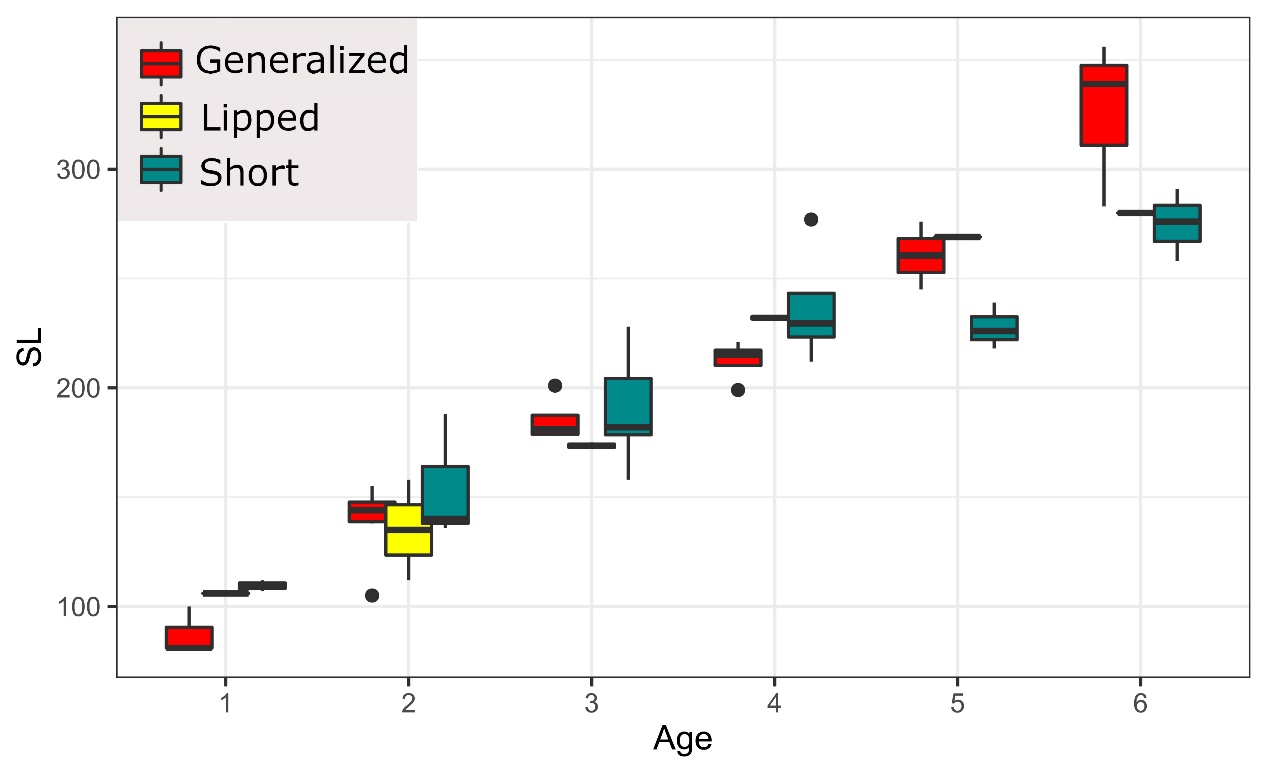

Supplement: S1 File — (DOCX) [file pone.0239639.s001.docx]
